# Dataset of acute repeated sessions of bifrontal transcranial direct current stimulation for treatment of intractable tinnitus: A randomized controlled trial

**DOI:** 10.1016/j.dib.2017.09.006

**Published:** 2017-09-13

**Authors:** Ali Yadollahpour, Arash Bayat, Samaneh Rashidi, Nader Saki, Majid Karimi

**Affiliations:** a Department of Medical Physics, School of Medicine, Ahvaz Jundishapur University of Medical Sciences, Ahvaz, Iran; b Bioelectromagnetic Clinic, Imam Khomeini Hospital, Ahvaz Jundishapur University of Medical Sciences, Ahvaz, Iran; c Musculoskeletal Rehabilitation Center, School of Rehabilitation, Ahvaz Jundishapur University of Medical Sciences, Ahvaz, Iran; d Hearing Research Center, Imam Khomeini Hospital, Ahvaz Jundishapur University of Medical Sciences, Ahvaz, Iran; e Department of Audiology, Iran University of Medical Sciences, Tehran, Iran

**Keywords:** Transcranial direct current stimulation, Acute stimulations, Tinnitus, Depression, Anxiety, DLPFC

## Abstract

Transcranial direct current stimulation (tDCS) has reportedly shown promising therapeutic effects for tinnitus (Forogh et al., 2016; Joos et al., 2014) [1], [2]. Studies are ongoing to determine optimum treatment protocol and the site of stimulation. Findings of the early studies are heterogeneous and most studies have focused on single session tDCS and short follow-up periods. There is no study on repeated sessions of tDCS with long term follow-up. This study presents the results of a randomized clinical trial investigating the therapeutic effects of acute multi-session tDCS over dorsolateral prefrontal cortex (DLPFC) on tinnitus symptoms and comorbid depression and anxiety in patients with chronic intractable tinnitus. The dataset includes the demographic information, audiometric assessments, tinnitus specific characteristics, and the response variables of the study. The response variables included the scores of tinnitus handicap inventory (THI), tinnitus loudness and tinnitus related distress based on 0–10 numerical visual analogue scale (VAS) scores, beck depression inventory (BDI-II) and beck anxiety inventory (BAI) scores. The dataset included the scores of THI pre and immediately post intervention, and at one month follow-up; the tinnitus loudness and distress scores prior to intervention, and immediately, one hour, one week, and at one month after the last stimulation session. In addition, the BDI-II, and BAI scores pre and post intervention are included. The data of the real (*n*=25) and sham tDCS (*n*=17) groups are reported. The main manuscript of this dataset is "Acute repeated sessions of bifrontal transcranial direct current stimulation for treatment of intractable tinnitus: a randomized controlled trial" (Bayat et al., submitted for publication) [3].

**Specifications Table**

**Table:** 

Subject area	Medicine
More specific subject area	Neurosciences and Otolaryngology, tinnitus
Type of data	Tables
How data was acquired	Audiometric assessments, clinical examinations, questionnaires including Tinnitus handicap inventory, numerical 0–10 visual analogue scale, Beck depression inventory (BDI-II) and beck anxiety inventory (BAI)
Data format	Raw, processed data, biosignals.
Experimental factors	A double blind randomized placebo controlled clinical trial investigated the therapeutic effects of acute multi-session tDCS on tinnitus symptoms and comorbid depression and anxiety in tinnitus patients with one month follow-up.
Experimental features	Auditory and clinical assessments were performed. Response variables were assessed prior intervention. The two groups of patients underwent real tDCS (n=25) and sham tDCS (n=17) consisting twice daily for 5 consecutive days (10 sessions) over DLPFC. After intervention the data were collected using different questionnaires up to one month follow-up.
Data source location	Imam Khomeini Hospital, Ahvaz, Iran, 31°18'11.5"N 48°44'41.9"E
Data accessibility	All of the data presented in this study are accessible within this article.

**Value of the data**

• Data presented in this paper are collected from a double blind randomized placebo controlled clinical trial with one month follow-up.
• This dataset presents the data on the effects of acute multisession tDCS on intractable chronic tinnitus with one month follow-up.
• The data consist of the tDCS effects on THI, tinnitus loudness and distress, as well as comorbid depression and anxiety as well as comprehensive demographic information, tinnitus characteristics and audiometric assessment.
• Using these data other researchers can perform advanced statistical analyses and modeling to shed more light on the efficacy and nature of tDCS efficacy in tinnitus treatment.
• The data can be used to develop treatment response predicting indexes.

## 1 Data

The data of this study were collected from a double blind randomized placebo controlled clinical trial investigated the therapeutic efficacy of tDCS in intractable chronic tinnitus (*n*=42). The data consist of two main categories including demographic information, tinnitus characteristics, and audiometric assessments of the patients and the second group data are the values of response variables at prior intervention and post intervention. The Table 1, Table 2 respectively present the demographic information, tinnitus characteristics, and audiometric assessments of the participants in the real tDCS and sham tDCS groups. Table 3 presents the response variables for prior and post treatment for the real tDCS. Table 4 represents these measures for the sham tDCS. For this group, only those patients who reported significant outcome in the last assessment were followed up.

**Table 1:** Demographic information, tinnitus characteristics, and audiometric assessments of the participants in the real tDCS.

**Patient**	**Age, *y***	**Sex**	**Quality**^a^	**Laterality**^b^	**Duration(y)**	**Hearing loss right/left**^c^
1	44	M	R	R	8	N/L
2	43	M	P	R>L	5	L/N
3	48	M	T	R<L	12	M/L
4	51	M	HPW	L	11	M/L
5	54	M	R	R	6	L/N
6	38	M	R	R=L	3	N/L
7	57	M	P	R>L	7	M/N
8	47	M	H	R	7	N/N
9	56	M	HPW	R	12	L/L
10	53	M	B	L	8	N/L
11	34	M	R	R	12	L/L
12	46	F	HPW	R	10	N/L
13	42	F	H	R=L	5	N/N
14	47	F	P	L	11	P/M
15	39	F	B	L<R	5	L/L
16	48	F	H U	R	8	M/L
17	46	F	B	L	10	N/M
18	33	F	H	R=L	9	N/N
19	43	F	P	R	6	N/N
20	45	F	R	R	4	N/N
21	60	F	R	R>L	12	N/M
22	50	F	R	R>L	7	L/L
23	55	F	H	R=L	8	M/L
24	46	F	B	R	5	L/P
25	42	F	R	R	4	L/N

Note: THI, Tinnitus Handicap Inventory.

a Tinnitus Quality codes: R, ringing; B, buzzing; H, hissing; T, ticking; HPW, high pitch whistling; P, pulsating.

b Tinnitus side: L, left; R, right, R=L, bilateral with no lateralization; R>L, bilateral lateralizing more to the right side; L>R, bilateral lateralizing more to the left side.

c Class of hearing loss; N normal hearing threshold (<20 dB), L mild hearing loss (20–40 dB), M moderate hearing loss (41–70 dB), S severe hearing loss (70–90 db), P profound hearing loss (>90 db).

**Table 2:** Demographic information, tinnitus characteristics, and audiometric assessments of the participants in the sham tDCS.

**No.**	**Age, *y***	**Sex**	**Quality**^a^	**Laterality**^b^	**Duration (*y*)**	**Hearing loss right/left**^c^
1	55	F	HPW	R	10	P/L
2	43	F	P	L	6	N/L
3	47	F	R+T	L	9	M/N
4	63	F	HPW	R<L	8	L/M
5	48	F	R	R>L	4	N/L
6	42	F	R	R	9	N/N
7	38	F	P	R	7	N/N
8	54	F	R+H	R=L	6	L/N
9	55	F	P	R	12	M/L
10	33	M	R	L	10	N/N
11	48	M	P	L	12	P/M
12	43	M	HPW	L	9	M/M
13	42	M	R	R<L	5	L/N
14	47	M	H+B	R	6	M/L
15	57	M	T	R>L	10	L/M
16	50	M	H	R	3	N/L
17	43	M	R	R>L	12	L/M

Note: THI, Tinnitus Handicap Inventory.

a Tinnitus Quality codes: R, ringing; B, buzzing; H, hissing; T, ticking; HPW, high pitch whistling; P, pulsating.

b Tinnitus side: L, left; R, right, R=L, bilateral with no lateralization; R>L, bilateral lateralizing more to the right side; L>R, bilateral lateralizing more to the left side.

c Class of hearing loss; N normal hearing threshold (<20 dB), L mild hearing loss (20–40 dB), M moderate hearing loss (41–70 dB), S severe hearing loss (70–90 db), P profound hearing loss (>90 db)

**Table 3:** The response variables for prior and post treatment for the real tDCS.

**THI**			**Loudness**					**Distress**					**BDI**		**BAI**	
**Pre**	**Post**	**Post-1m**	**Pre**	**Post-i**	**Post-1h**	**Post-1w**	**Post-1m**	**Pre**	**Post-i**	**Post-1h**	**Post-1w**	**Post-1m**	**Pre**	**Post**	**Pre**	**Post**
67	40	44	8	5	5	7	7	8	6	6	7	8	25	11	23	12
65	38	41	7	5	5	6	6	8	6	6	6	7	22	10	26	10
63	65	60	8	9	9	9	8	8	9	9	9	9	25	12	25	15
73	62	65	9	9	9	9	9	8	8	8	8		24	18	24	16
85	40	43	8	5	5	5	7	9	7	7	8	8	25	14	22	9
75	45	46	7	4	4	5	5	7	5	5	6	6	27	13	23	18
82	50	60	9	7	7	7	9	8	7	7	7	8	26	12	28	15
57	35	32	7	4	4	4	6	8	6	6	7	7	24	11	23	16
66	40	43	8	5	5	5	6	7	5	5	6	6	28	11	21	9
54	25	32	6	4	4	4	5	6	5	5	5	6	25	13	19	11
81	47	64	9	7	7	7	9	8	7	7	7	8	26	12	24	13
89	60	58	8	6	6	6	7	8	6	6	7	7	28	12	26	16
74	42	47	6	4	4	5	5	7	5	5	6	6	22	12	26	12
63	55		7	8	8	8	8	8	8	8	8	8	24	20	25	21
65	33	37	6	4	4	4	5	7	5	6	6	6	27	19	26	21
85	50	55	7	4	4	4	5	7	6	6	6	7	26	12	29	11
63	58	60	6	7	7	6	6	8	8	8	8	8	34	14	28	16
78	75	70	7	7	7	7	7	8	8	8	8	8	22	11	20	9
66	31	36	8	5	6	6	7	8	6	7	7	7	29	12	29	14
64	42	44	7	5	5	6	6	8	6	7	7	7	23	11	24	10
74	48	55	7	5	5	6	6	8	7	7	8	8	30	14	32	18
61	37	40	7	5	5	5	6	7	5	5	5	6	25	11	24	11
58	55	54	8	9	9	8	8	8	8	8	8	8	32	27	30	20
72	68	70	9	9	9	9	9	8	7	7	8	8	15	12	21	15
67	40	45	7	5	5	5	6	8	6	6	6	7	24	10	26	13

Note: For the treatment assessments, only those patients who reported significant outcome in the last assessment were followed up. Pre: pre-intervention, post: post-intervention, post-i: immediately after intervention, post-1h: at one hour post intervention; post-1w: at one week post intervention; post-1m: at one month post intervention. Tinnitus loudness and distress ranged 0–10 where 0 indicates the lowest level and 10 indicates the highest tolerable level.

**Table 4:** The response variables for prior and post treatment for the sham tDCS.

**THI**			**Loudness**					**Distress**					**BDI**		**BAI**	
**Pre**	**Post**	**Post-1m**	**Pre**	**Post-i**	**Post-1h**	**Post-1w**	**Post-1m**	**Pre**	**Post-i**	**Post-1h**	**Post-1w**	**Post-1m**	**Pre**	**Post**	**Pre**	**Post**
81	75	–	8	8	8	8	–	8	7	8	8	–	22	18	22	19
67	54	–	7	6	7	7	–	8	8	8	8	–	25	19	26	18
70	72	–	8	8	8	8	–	8	8	8	8	–	24	22	25	20
75	70	–	8	8	8	8	–	8	8	7	8	8	23	21	22	20
66	44	68	7	6	6	7	7	8	7	8	8	–	22	18	27	25
70	69	–	9	9	9	9	–	8	8	8	8	–	22	27	28	21
75	54	–	7	6	6	7	7	8	7	7	8	8	24	14	22	12
56	50	–	7	7	7	7	–	8	8	8	8	–	19	15	16	14
72	65	–	8	8	8	8	–	7	7	7	7	–	28	25	27	23
54	50	–	7	6	7	7	–	6	5	6	6	–	25	20	24	19
67	65	–	9	9	9	9	–	8	9	9	9	–	28	26	28	27
86	84	–	8	7	8	8	–	8	8	8	8	–	18	22	23	17
78	70	–	6	5	6	6	–	7	5	6	6	–	19	21	25	14
83	76	–	7	7	7	7	–	8	7	8	8	–	29	28	24	20
66	60	–	6	6	6	6	–	7	6	7	7	–	22	13	24	13
54	30	55	8	7	7	8	8	7	5	5	6	6	28	20	27	18
67	65	–	9	9	9	9	–	8	9	9	9	–	28	26	23	21

Note: For the treatment assessments, only those patients who reported significant outcome in the last assessment were followed up. Post-i: immediately after intervention, post-1h: at one hour post intervention; post-1w: at one week post intervention; post-1m: at one month post intervention. Tinnitus loudness and distress ranged 0–10 where 0 indicates the lowest level and 10 indicates the highest tolerable level.

## 2 Experimental design, materials and methods

### 2.1 Study design

In a double blinded randomized controlled clinical trial, patients with chronic intractable tinnitus (*n*=42) were divided into two groups of real (females (*F*)=14, males (*M*)=11, age: 46.68±6.87 years, disease duration: 7.8±2.84 years), and sham tDCS (*F*=9, *M*=8, age: 47.53±7.56 years, disease duration: 8.11±2.8 years). Both groups were matched for age, gender, ethnicity, and audiometric main characteristics. To reduce the procedure and subjective bias, the patients, the researchers who collected the data, and the researchers who performed statistical analyses and interpretations were blinded on the type of protocol. Before the intervention, the patients underwent complete audiometric and neurologic examinations by experienced specialists. The experimental procedures of the present study including tDCS sessions, outcomes evaluations were performed in the Bioelectromagnetic Clinic in Ahvaz Imam Hospital, an affiliated Hospital to Ahvaz Jundishapur University of Medical Sciences (AJUMS), Iran. All of the experimental procedures of this study were approved by the local ethics committee of AJUMS, Ahvaz, Iran (registration code: IR.AJUMS.REC.1394.639) which were in accordance with the ethical standards and regulations of human studies of the Helsinki declaration (2014) [4]. All participants filled and signed a written consent form for contribution in the study following a clearly description of the experimental procedures, the objectives, possible benefits, and side effects of the study to the patients. The study was registered as a clinical trial in the Iranian registry of clinical trials (IRCT2016110124635N5).

### 2.2 Hearing assessment

Pure-tone audiometry was performed using AC 40 dual channel Audiometer (Intracoustics Co., Denmark). The hearing thresholds were recorded over the frequency ranges of 250 to 8000 Hz for air conduction and 500 to 4000 Hz for bone conduction pathways, using the modified Hughson–Westlake Method as recommended by ANSI 2005 [5]. Pure-tone audiometry was considered normal when the hearing thresholds at all frequencies were below 20 dBHL. Hearing loss was classified according to the type and degree [6].

### 2.3 tDCS intervention

The tDCS was performed through carbon electrodes embedded in a saline-soaked pair of sponges (35 cm^2^) and delivered by a battery-driven, constant current stimulator with a maximum output of 4 mA (OASIS Pro^™^ device by Mind Alive Inc., Edmonton, Alberta, Canada). The real tDCS consisted of twice daily sessions (intersession interval of 6 hours) of 2 mA current for 20 min for 5 consecutive days through 35 cm^2^ electrodes. The anode was placed over the right DLPFC (F4), and the cathode over the left DLPFC (F3). In the sham group, the electrode montage was identical, but the device was turned off after 30 s without the participant's knowledge. The THI was assessed pre and post intervention, and at one month follow-up. Tinnitus loudness and distress were scored using a 0–10 rating numerical visual analogue scale prior to intervention, and immediately, one hour, one week, and at one month after the last stimulation session. Depression and anxiety scores were determined pre and post intervention using the Beck Depression (BDI-II) and Anxiety Inventories (BAI). Moreover, blinding quality of the study was assessed. During treatment, the patients were asked to remove all metal-based jewelry from the head and neck.

### 2.4 Clinical evaluation

The tinnitus quality for each patient was determined through asking the patient and confirmed by the otolaryngologist/audiologist experts. The tinnitus was categorized as ringing, buzzing, hissing, humming, ticking, high pitch whistling, thumping, cicadas, and pulsating.

The main measures or response variables included THI, tinnitus loudness and distress, and depression and anxiety. The THI was assessed pre and post intervention, and at one-month follow-up. Tinnitus loudness and distress were scored using a 0–10 rating numerical visual analogue scale (VAS) prior to intervention, and immediately, one hour, one week, and at one month after the last stimulation session. Depression and anxiety scores were determined pre and post intervention using the BDI-II and BAI.
